# A Method of Generating and Administering Medicated Steam to Horses1Read before the Missouri Valley Veterinary Association, June 8, 1898.

**Published:** 1898-08

**Authors:** R. C. Moore

**Affiliations:** Kansas City, Mo.


### A METHOD OF GENERATING AND ADMINISTERING MEDICATED STEAM TO HORSES.[1]

By R. C. Moore, D.V.S.,
KANSAS CITY, MO.

I PRESUME every practising veterinarian is familiar with the advantages derived from inhalations of medicated steam in the treatment of acute and chronic inflammation of the air-passages.

The difficulty in thorough administration has caused me to pass it by many times when I am sure it would have done good service.

In May, 1896, I had serious trouble with some cases of contagious pleuro-pneumonia in horses (or I think, more properly speaking, necrotic pneumonia), several cases of which terminated fatally.

On visiting the stable one morning at that time I found two more cases that would soon go the same road. I determined to try treatment by medicated steam, but how was I to generate the steam and how to administer it to the horse effectually.

On looking around the place for some available method to accomplish this I found a small gasoline-stove; this could be made to supply the heat. Next I found an oil-can (capacity one gallon) fitted at the top to receive a cork; this would hold the water and

[1] Read before the Missouri Valley Veterinary Association, June 8, 1898.

medicine. So the task of generating the steam was mastered. Next I found a piece of garden-hose ten or twelve feet long, the end of which would fit into the socket in the top of the can; this would convey the steam from the boiler to the horse; but how should I get the horse to inhale it? A heavy canvas grain-bag was at hand; the open end of this secured around the horse's nose up nearly to the eyes, by means of a cord over his head and a hole in the bottom through which the end of the hose was inserted and tied, answered this purpose and completed the rig.

One quart of water, one ounce each of fl. ext. bell. and hydrastis cand. were placed in the can, and it placed over the lighted fire; when boiling the hose was attached and the steaming continued twenty-five to thirty minutes, and repeated once or twice daily, according to the urgency of the symptoms. After this we lost no more cases of this form of pneumonia, and it has proved equally serviceable in all throat and bronchial troubles.

I do not mean that everyone should adopt these particular appliances, for a properly-constructed rubber or ducking nose-bag would be far better, and a differently-constructed boiler with protruding spout, over which the hose could be slipped, would also be an improvement.

The advantage, it seems to me, in this method over a pail of hot water, scalded feed, etc., is the quantity of steam one can generate and the continuance of its administration. For by continuing it for one-half hour, where every bit of air the horse inhales is thoroughly charged with the medicine, it reaches every spot of diseased surface, and the several repeated applications must surely be of more service than one can possibly get from the old method.

